# Staging and Grading Advanced Periodontitis with Large Language Models from Standardized Case Summaries: A Multicenter Concordance and Reproducibility Study

**DOI:** 10.3390/jcm15197585

**Published:** 2026-09-30

**Authors:** Mehmet Kızıltoprak, Mustafa Karaca, Mustafa Özay Uslu

**Affiliations:** 1 Department of Periodontology, Faculty of Dentistry, Batman University, 72060 Batman, Türkiye; mehmet.kiziltoprak@batman.edu.tr; 2 Department of Periodontology, Faculty of Dentistry, Burdur Mehmet Akif Ersoy University, 15030 Burdur, Türkiye; mustafakaraca@mehmetakif.edu.tr; 3 Department of Periodontology, Faculty of Dentistry, Alanya Alaaddin Keykubat University, 07410 Alanya, Türkiye

**Keywords:** artificial intelligence, diagnostic concordance, large language models, periodontitis, staging and grading, reproducibility

## Abstract

**Background/Objectives**: To evaluate the concordance of three general-purpose large language models (LLMs), ChatGPT, Claude, and Gemini, with an adjudicated expert consensus classification of Stage, Grade, and Extent in Stage III–IV periodontitis when applying the 2018 American Academy of Periodontology/European Federation of Periodontology (AAP/EFP) rules to clinician-prepared, standardized case summaries. **Methods**: In this multicenter retrospective concordance study (two centers, 105 patients), two periodontologists independently classified each case from complete charts and radiographs; discordant cases were adjudicated. De-identified summaries containing pre-extracted clinical, radiographic, and modifier variables were presented to each LLM in three independent sessions per case; the modal decision was the primary index result, and single-session performance was also reported. Stage and Grade concordance (Cohen’s/weighted kappa) were co-primary endpoints; Extent and full concordance were secondary. **Results**: Modal Stage concordance was almost perfect for ChatGPT (κ = 0.98) and Gemini (κ = 0.92) and substantial for Claude (κ = 0.73); single-session Stage κ ranged from 0.73 to 0.98. Grade (weighted κ = 0.97–1.00) and Extent (κ = 0.93–1.00) concordance were almost perfect. Full concordance ranged from 85.7% (Claude) to 99.0% (ChatGPT). Every classifiable modal Stage discordance understated severity: Claude and Gemini assigned Stage II or I to 14 Stage III cases in which attachment loss met the Stage III threshold but radiographic bone loss was below 33%. **Conclusions**: Given pre-extracted variables and explicit instructions, the LLMs applied the classification rules with substantial to almost perfect concordance; this reflects rule application, not independent diagnosis. Performance was model-specific, Gemini showed greater session-to-session variability, and two models systematically understaged borderline Stage III cases. The findings support only clinician-supervised, educational use with local validation.

## 1. Introduction

Accurate staging, grading, and extent determination per the 2018 American Academy of Periodontology/European Federation of Periodontology (AAP/EFP) classification [1,2] underlies subsequent periodontal treatment decisions, reflected in stage-specific EFP S3 guidelines [3,4]. The framework is not a simple lookup: Stage integrates clinical attachment loss, radiographic bone loss, tooth loss attributable to periodontitis, and case complexity factors, while Grade layers historical rate of progression together with risk modifiers such as smoking and diabetes onto this severity assessment, and Extent requires a separate judgment about the proportion of affected sites. Because the framework integrates clinical, radiographic, and modifier-based criteria across several weighted dimensions, its application remains imperfect even among specialists [5,6], with disagreement concentrated at the boundaries between adjacent stages and grades, where multiple criteria must be weighed jointly rather than read off a single measurement.

General-purpose large language models (LLMs) such as ChatGPT, Claude, and Gemini have moved rapidly into clinical and educational use across medicine and dentistry, raising the question of whether they can support, or eventually approximate, tasks that have traditionally required specialist judgment. In periodontology specifically, the uptake of these tools has prompted growing evaluation of periodontal knowledge, examinations, treatment planning, and guideline extraction [7,8,9,10,11,12]. Tastan Eroglu et al. [13] found ChatGPT reproduced Stage/Grade for 200 real patients with only fair-to-poor agreement (kappa ≈ 0.20–0.30), whereas a reinstructed GPT-4o [14] and GPT-5 [15] performed better when given more explicit guideline-based instructions. LLM output can also be inconsistent across repeated sessions [16,17], and because no LLM has been formally validated as a periodontal diagnostic aid, concordance is interpretable only against a human benchmark, a comparison complicated by the fact that international panels show meaningful disagreement even among trained examiners applying the same criteria [5,6].

Several questions remain open for advanced periodontitis specifically, where correct Stage and Grade assignment carries the greatest treatment consequences and where the 2018 AAP/EFP criteria are most complex to apply. Reported concordance has varied widely across studies, from fair-to-poor to substantial, and this variation is difficult to interpret without knowing whether the reference classification reflected an independently adjudicated consensus or a single reviewer’s judgment, whether more than one model or testing round was used, and whether the same case was tested only once or repeatedly. Session-to-session reproducibility, an essential property for any tool intended for repeated clinical or educational use, has rarely been assessed alongside accuracy. To our knowledge, no prior study has combined real, multicenter patient records, an independently adjudicated expert consensus, and repeated, memory-disabled sessions across several widely used models for advanced (Stage III–IV) periodontitis. We chose ChatGPT, Claude, and Gemini as the most widely used public general-purpose LLM platforms at the time of study design, aiming to compare leading commercial ecosystems rather than to be exhaustive.

The aim was therefore to evaluate, in real patients with Stage III or IV periodontitis, the extent to which ChatGPT, Claude, and Gemini, given standardized clinical and radiographic summaries and an explicit 2018 AAP/EFP instruction, reproduce the Stage, Grade, and Extent assigned by an adjudicated expert consensus based on complete periodontal charting and raw panoramic radiography. Because the models received variables that clinicians had already extracted and structured, the study evaluates the application of classification rules to prepared data rather than independent periodontal diagnosis; a secondary aim was to quantify session-to-session reproducibility and to characterize the type and direction of classification errors.

## 2. Materials and Methods

### 2.1. Study Design, Setting, and Reporting

This was a multicenter retrospective concordance study; the adjudication procedure, LLM evaluation protocol, and statistical plan were prespecified before the dataset was locked. This study was reported primarily in accordance with STROBE and GRRAS, with relevant items from STARD-AI and TRIPOD-LLM incorporated where applicable [18,19,20,21,22]; checklists, the system instruction, and case-form template are Supplementary Files. Ethical approval for the use of records from both participating centers was granted by the Non-Interventional Clinical Research Ethics Committee of Burdur Mehmet Akif Ersoy University (no. GO 2026/3358, 7 July 2026). Following this approval, separate institutional permission was obtained from Batman University for access to records at the Batman center. Only standardized, de-identified summaries were presented to the LLMs; the reference standard is termed the adjudicated expert consensus reference classification rather than a “gold standard”.

### 2.2. Participants

Records were drawn from two periodontology clinics (Center A, Batman; Center B, Burdur) following ethics approval (7 July 2026). All consecutive patients who had attended either clinic between February 2025 and June 2026 with a screening diagnosis of Stage III or IV periodontitis were screened for eligibility; no quota sampling or selection by case difficulty was applied. Records were collected and summaries prepared 8–18 July 2026, preceding the 18–24 July 2026 LLM testing window (Section 2.4). Eligible patients were ≥18 years with a screening diagnosis of Stage III or IV periodontitis, complete periodontal charting, a same-day panoramic radiograph, and adequate history; exclusions were antibiotics within 6 months, incomplete records, prior periodontal surgery, active oncologic disease/bisphosphonate therapy, or pregnancy, and Stage I–II periodontitis was excluded a priori. Accrual ended when all eligible records within this period had been screened, and the dataset was locked on 18 July 2026, before any LLM session was run; the final sample of 105 therefore reflects the number of eligible records available rather than a stopping rule based on results. Screening diagnosis was chart-based; the reference Stage was established independently via consensus reached after adjudication (Section 2.3), avoiding circularity with the outcome measure.

### 2.3. Reference Standard: Adjudicated Expert Consensus

Two center-based periodontologists (Experts A and B; 7 and 8 years of post-certification experience) independently reviewed each complete chart and radiograph in Round 1 while blinded to LLM output. Overall inter-rater agreement was assessed using Cohen’s kappa for Stage and Extent and linear-weighted kappa for Grade; a kappa below 0.60 would have prompted re-calibration of Experts A and B, but the observed agreement exceeded this threshold. At the individual case level, Round 1 classifications were compared to identify discordant cases (Round 2). These discordant cases entered structured consensus (Round 3) between Experts A and B; consensus was reached for 5 of these cases, and the remaining 7 discordant cases were referred to binding adjudication (Round 4) by a third periodontologist (Expert C; 12 years of post-doctoral experience), who had not taken part in Round 1 and joined the study for this adjudicating role. Expert C reviewed only the same standardized, de-identified case summaries used for the LLM evaluation (Section 2.4), did not access identifiable patient records or raw radiographs, and was blinded to LLM output. This restriction was adopted for data-protection reasons, because Expert C was based at a third institution not covered by the record-access permissions; we acknowledge that it means the reference standard for these 7 cases (6.7%) was formed from a narrower information base than for the remaining 98 cases. To examine whether this affected the results, all concordance analyses were repeated with these 7 cases excluded, and additionally with each Round 1 expert’s independent classification used directly as the reference (Section 3.8). The resulting decisions formed the final reference classification (Round 5).

### 2.4. Index Tests: Large Language Models

We evaluated ChatGPT (OpenAI, San Francisco, CA, USA), Claude (Anthropic, San Francisco, CA, USA), and Gemini (Google, Mountain View, CA, USA) through their standard consumer websites (chatgpt.com, claude.ai, and gemini.com). Default settings were retained, and neither application programming interface (API) access nor custom tuning was used, reflecting ordinary end-user access. From 18 to 24 July 2026, every case was tested in three separate sessions per model, each in a new conversation with memory disabled, at least 48 h apart, and in randomized order. We recorded the platform, testing window, and the model label displayed in the user interface for every session (Section 3.3); no displayed label changed during testing. These labels are reported exactly as displayed by each platform at the time of testing; they are consumer-facing designations, and the vendors do not disclose whether the underlying model weights or serving configuration behind a given label remain fixed. An unchanged label therefore does not guarantee an unchanged backend model (Section Limitations). The findings therefore apply specifically to the recorded labels (ChatGPT GPT-5.6 Sol, Claude claude-sonnet-5-medium, and Gemini 3.1 Pro) and not necessarily to other versions or deployment settings.

Each model received an unmodified system instruction to act as a periodontology assistant, answer using only the supplied data, and classify strictly per the 2018 AAP/EFP framework [2], including the alternative Stage IV criteria (≥1 complexity factor, including posterior bite support loss, pathological migration, or masticatory dysfunction, or ≥5 teeth lost). Models gave a one-sentence final decision and ≤2-sentence rationale (exact wording in File S4), derived directly from the 2018 AAP/EFP criteria without a separate development dataset or pilot-testing. The case form presented demographic/systemic data, charting and radiographic summaries (Section 2.6), grade modifiers, and complexity factors; Extent was computed from affected/remaining tooth counts.

### 2.5. Blinding and Separation of Data Streams

The researchers who prepared the case summaries and conducted the model sessions did not know the reference classification. The researcher summarizing radiographic findings was also unaware of the consensus result, and separate data-entry sheets kept LLM outputs from the experts. No researcher conducted all three sessions for a case-model pair. Because every response ended with an explicit Stage, Grade, and Extent decision (File S4), decisions were transcribed verbatim. A response without a clear final decision would have been coded unclassifiable (Section 2.7).

### 2.6. Extent Determination and the Expert–LLM Information Asymmetry

Extent was classified as localized when no more than 30% of the remaining teeth were affected and as generalized when the proportion exceeded 30%, per the 2018 AAP/EFP framework [2]. Experts used the complete chart; models used the same affected- and remaining-tooth counts provided in the summary. Unlike the experts, the LLMs did not inspect the original record or radiograph. More generally, the case form supplied Grade modifiers (smoking, HbA1c) and the three Stage IV complexity factors as pre-coded categories or binary flags, and tooth loss attributable to periodontitis as a count, so that these components of the classification required little more than a look-up against the stated rules. The Stage decision, by contrast, still required the model to integrate maximal interdental clinical attachment loss (CAL), percentage radiographic bone loss (RBL), tooth loss, and complexity factors under the framework’s compound rules. This deliberate design isolates rule application to standardized summaries from data extraction and image interpretation; it also means the study cannot distinguish a model’s intrinsic capability from the simplification of the task produced by pre-structuring the inputs (Section Limitations).

### 2.7. Outcomes and Decision Rules

Stage concordance, assessed with Cohen’s kappa, and Grade concordance, assessed with linear-weighted kappa for the ordered A/B/C categories, were the co-primary outcomes (Bonferroni α = 0.025 each). Secondary outcomes were Extent concordance (Cohen’s kappa, α = 0.05) and full concordance, defined as simultaneous agreement with the reference on Stage, Grade, and Extent. The primary analyses used the modal decision across the three sessions (below). Because a clinician or student would ordinarily query a model once, single-session concordance for each of the three sessions was also reported for the same endpoints, and the number of cases with at least one discordant session was recorded. Exploratory analyses examined stable errors, session instability, the vote pattern across sessions (3/3 unanimous, 2/1 split, or 1/1/1 three-way) as an indicator of decision stability, model labels, and center effects.

A modal decision was defined before the analyses for each case-model pair. Three identical responses constituted a consistent decision, whereas a two-to-one split was resolved by the majority response. A three-way split was labeled ‘no stable modal decision’ and counted as discordant, with the instability also reported separately. Responses without a usable classification were to be labeled unclassifiable and counted as discordant. Final coding was binary, either concordant or discordant, and did not award partial credit.

### 2.8. Sample Size

We determined the sample size from the expected precision of kappa rather than from a fixed hypothesis test; the calculation targeted the precision of the prespecified kappa estimates rather than statistical power for a particular null-hypothesis test, so no β-based power calculation was used. Tastan Eroglu et al. [13] reported Stage kappa values of approximately 0.20–0.30. Under a target kappa of 0.40 with a lower bound of 0.20, at least 45 cases were required; a conservative target of 0.30 with a lower bound of 0.10 required 58. Together with a prespecified pilot rule, these estimates supported a minimum of 60 (full derivation in File S4). The pilot rule, applied to the first 10 consecutively accrued cases as an internal pilot, could only have increased the minimum sample (to 80 or 100); the observed pilot value (Fleiss’ κ = 1.00 across the three models’ modal Stage decisions) left the minimum at 60. All eligible consecutive records from the accrual period (Section 2.2) were included; accrual continued until the dataset was locked at 105 cases, which exceeded the largest sample that the pilot rule could have required (*n* = 100). The sample size was not planned for between-model comparisons; all such comparisons are exploratory and unadjusted for multiplicity (Section 2.9). The final Stage distribution (67.6% Stage III and 32.4% Stage IV) and Grade distribution (6.7% A, 41.0% B, and 52.4% C) arose from the eligible records at the two centers rather than from quota sampling.

### 2.9. Statistical Analysis

We used Cohen’s kappa for Stage and Extent and linear-weighted kappa for Grade, so that a Grade A–C disagreement carried more weight than an A–B disagreement; linear rather than quadratic weights were used because the three-category ordinal scale does not justify a squared distance penalty. Values were interpreted using Landis and Koch [23] benchmarks (<0.00 poor; 0.81–1.00 almost perfect). Confidence intervals (CIs) came from 10,000 patient-level percentile bootstrap samples because Wald intervals may exceed the upper limit near perfect agreement. Co-primary outcomes use 97.5% CIs (α = 0.025); other outcomes use 95% CIs. Fleiss’ kappa [24] was used for reproducibility across sessions, and radiographic reliability was summarized with the intraclass correlation coefficient (ICC(2,1)) [25]. Exploratory analyses included Cochran’s Q for variation in session-level concordance, the direction of Stage and Grade errors (understaging/undergrading or overstaging/overgrading), and the distribution of vote patterns across sessions in relation to concordance.

A prespecified exploratory Bayesian mixed-effects logistic regression was fitted to session-level Stage concordance (945 sessions). Fixed effects were model (reference: ChatGPT), reference Stage (reference: Stage III), and center (reference: Center B), with a random intercept per patient to account for the clustering of nine sessions within each case. Weakly informative priors were used: Normal (0, 2.5) on the intercept and all fixed-effect coefficients (logit scale) and Half-Normal (2.5) on the standard deviation of the patient random intercept. Posteriors were sampled with the No-U-Turn Sampler (4 chains, 2000 warm-up and 2000 sampling iterations each, target acceptance 0.95) in PyMC 5.28 via Bambi 0.17. Convergence was assessed by the potential scale reduction factor (R-hat), bulk and tail effective sample sizes (ESS), and the number of divergent transitions. Results are posterior median odds ratios (ORs) with 95% credible intervals (CrIs). Because Stage distribution differed between centers, three additional specifications were fitted to examine the collinearity of these two effects: without the Stage term, without the center term, and with a Stage × center interaction. Because ChatGPT produced only three discordant sessions, the between-model ORs are imprecise and should be viewed as exploratory; between-model comparisons were not adjusted for multiplicity. The model specification and code are given in File S4. Analyses were performed in Python 3.11 using pandas 3.0, scikit-learn 1.8 (kappa statistics), SciPy 1.17, PyMC 5.28, Bambi 0.17, and ArviZ 0.23.

### 2.10. Use of Artificial Intelligence in Manuscript Preparation

The authors wrote the manuscript. After the authors had completed the draft, Claude (claude-sonnet-5-medium; Anthropic, San Francisco, CA, USA; accessed via claude.ai) was used only to proofread the English and improve language clarity. All authors reviewed the edited text and approved the final wording. The tool had no role in the study design, data collection or analysis, interpretation of the findings, or creation of original scientific content. The authors verified all analyses and statements and accept full responsibility for the accuracy and integrity of the manuscript.

## 3. Results

### 3.1. Patient and Case Characteristics

**Figure 1:**
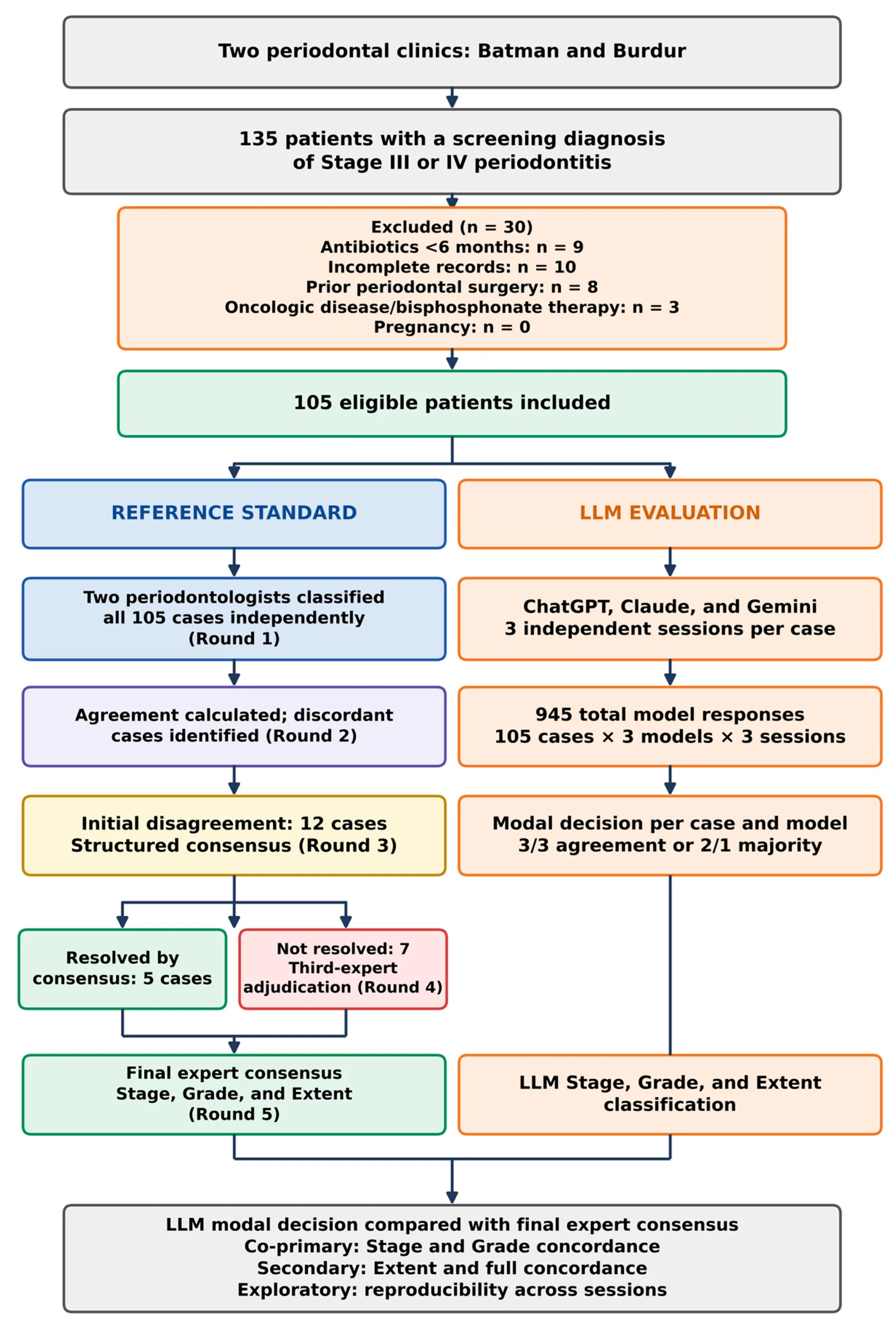
Study flow. Of 135 patients with a screening diagnosis of Stage III or IV periodontitis identified at the two centers, 30 were excluded (antibiotics within 6 months, *n* = 9; incomplete records, *n* = 10; prior periodontal surgery, *n* = 8; active oncologic disease or bisphosphonate therapy, *n* = 3; pregnancy, *n* = 0), leaving 105 eligible patients who were all included. Records were then evaluated in parallel. For the reference standard, two periodontologists classified all cases independently; the 12 cases with initial disagreement underwent structured consensus, which resolved 5 cases; the remaining 7 cases were adjudicated by a third, independent periodontologist. The index tests comprised three large language models (LLMs): ChatGPT, Claude, and Gemini, each queried in three independent sessions per case. The modal LLM decision per case was compared against the adjudicated expert consensus reference classification in the concordance analysis. *N*, number of cases; LLM, large language model.

Of 135 screened patients, 30 were excluded: 9 had received antibiotics within the previous 6 months, 10 had incomplete records, 8 had undergone periodontal surgery, and 3 had oncologic disease or bisphosphonate therapy; none were pregnant. The final sample comprised 105 patients (Figure 1, Table 1): 50 from Center A in Batman (47.6%) and 55 from Center B in Burdur (52.4%). Mean age was 56.8 ± 11.9 years (range, 29–84), and 61 patients (58.1%) were male. A total of 46 patients (43.8%) smoked, 1 (1.0%) was a former smoker, and 10 (9.5%) had diabetes. The reference assigned 71 cases (67.6%) to Stage III and 34 (32.4%) to Stage IV. Grade A, B, and C accounted for 7 (6.7%), 43 (41.0%), and 55 (52.4%) cases, respectively. Extent was localized in 16 cases (15.2%) and generalized in 89 (84.8%).

### 3.2. Reference Standard Reliability and Radiographic Measurement Reliability

Before adjudication, the two experts agreed substantially on Stage (κ = 0.79) and almost perfectly on Grade (weighted κ = 0.91) and Extent (κ = 1.00). Thus, the prespecified threshold of κ < 0.60 was not met. Their complete three-dimensional classifications matched in 93 of 105 cases (88.6%). The other 12 cases (11.4%) entered the structured consensus round, which resolved 5 cases (4.8%); the remaining 7 cases (6.7%) were referred to and resolved by third-expert adjudication (Section 2.3). Agreement between the paired radiographic bone-loss measurements was also high (ICC(2,1) = 0.97, 95% CI 0.95–0.98; *n* = 105 pairs).

### 3.3. Model Testing Characteristics

Testing produced 945 LLM sessions: 105 cases assessed three times by each of three models, or 315 sessions per model. For each platform, the displayed model label remained unchanged from 18 to 24 July 2026 (Table 2); whether the underlying model remained unchanged cannot be verified (Section Limitations).

### 3.4. Primary Endpoint 1: Stage Concordance

Stage agreement was almost perfect for ChatGPT (κ = 0.98, 97.5% CI 0.91–1.00; 99.0% agreement) and Gemini (κ = 0.92, 97.5% CI 0.82–1.00; 96.2% agreement), but lower, although still substantial, for Claude (κ = 0.73, 97.5% CI 0.59–0.86; 85.7% agreement) (Table 3). ChatGPT disagreed with the reference once, assigning Stage III to a Stage IV case. Among Claude’s 15 discordant cases, 12 were Stage III cases assigned Stage II, 1 was a Stage III case assigned Stage I, 1 was a Stage IV case assigned Stage III, and 1 had no stable modal decision; 7 of the 15 received the same incorrect Stage in all three sessions and 7 arose from an incorrect two-to-one split. Gemini’s 4 discordant cases comprised 3 Stage III cases assigned Stage I and 1 Stage IV case assigned Stage III. Thus, every classifiable modal Stage discordance understated severity, and four modal decisions (Claude 1, Gemini 3) were two categories below the reference (Section 3.8). No response from any model was unclassifiable.

### 3.5. Primary Endpoint 2: Grade Concordance

For Grade, agreement was almost perfect across all three models (ChatGPT weighted κ = 1.00; Claude and Gemini both weighted κ = 0.97, 97.5% CI 0.91–1.00) (Table 4). Claude made two errors, both assigning Grade B instead of A. Gemini made two errors: one undergrading from B to A and one overgrading from B to C. The Gemini B-to-A decision was the only modal undergrading error observed. All four Grade discordances occurred in cases with a two-to-one split across sessions; no Grade error occurred in a case with three identical responses (Section 3.7).

### 3.6. Secondary Endpoints: Extent and Full Three-Dimensional Concordance

ChatGPT and Claude matched the reference for Extent in every case (κ = 1.00). Gemini’s Extent agreement was almost perfect (κ = 0.93, 95% CI 0.80–1.00) (Table 5). Gemini’s two Extent discordances (one localized case classified as generalized and one generalized case classified as localized) both occurred in cases with a two-to-one split across sessions. When Stage, Grade, and Extent all had to match simultaneously, concordance was 99.0% for ChatGPT (104/105, 95% CI 97–100%), 95.2% for Gemini (100/105, 95% CI 91–99%), and 85.7% for Claude (90/105, 95% CI 79–92%).

### 3.7. Reproducibility Across Sessions and Single-Session Performance

Table 6 cross-tabulates the vote pattern across the three sessions against modal concordance. ChatGPT gave three identical Stage responses in every case (104 concordant, 1 discordant). Claude gave three identical responses in 92 cases (85 concordant, 7 discordant), a two-to-one split in 12 cases (5 concordant, 7 discordant), and a three-way split in 1 case. Gemini gave three identical responses in 90 cases (89 concordant, 1 discordant) and a two-to-one split in 15 (12 concordant, 3 discordant). A split vote therefore marked a substantially higher probability of error: for Stage, 7 of 12 split cases (58%) versus 7 of 92 unanimous cases (7.6%) were discordant for Claude, and 3 of 15 (20%) versus 1 of 90 (1.1%) for Gemini. For Grade and Extent, every discordance across all models occurred in a split case and none in a unanimous case. Nevertheless, 7 of Claude’s Stage errors were unanimous across sessions; repeated querying cannot detect an error that a model makes consistently.

ChatGPT was the most reproducible model across the three sessions, with Fleiss’ kappa values of 1.00 for Stage, 0.99 for Grade, and 1.00 for Extent (Table 2). Reproducibility remained in the almost-perfect range for Claude (Stage 0.85, Grade 0.91, Extent 1.00) and Gemini (Stage 0.81, Grade 0.89, Extent 0.85). Nevertheless, Cochran’s Q identified significant between-session variation for Gemini (Section 3.8).

Table 7 reports concordance for each session separately, i.e., the performance a user would obtain from a single query. For ChatGPT, single-session values were indistinguishable from the modal results (Stage κ = 0.98 in every session; full concordance 98.1–99.0%). For Claude, single-session Stage κ ranged from 0.73 to 0.75 and full concordance from 84.8% to 86.7%, again close to the modal values, indicating that its lower concordance reflected stable rather than random error. For Gemini, the modal rule materially improved apparent performance: single-session Stage κ was 0.81 in sessions 1 and 3 but 0.98 in session 2, Extent κ ranged from 0.81 to 1.00, and full concordance ranged from 85.7% to 99.0%, compared with 95.2% for the modal decision. At least one discordant session (any dimension) occurred in 2 of 105 cases for ChatGPT, 23 for Claude, and 23 for Gemini.

### 3.8. Exploratory Analysis: Error Type, Error Direction, Decision Stability, and Reference-Standard Sensitivity

In exploratory, multiplicity-unadjusted analyses, Cochran’s Q did not indicate session differences for ChatGPT (Grade: Q = 2.0, nominal *p* = 0.37) or Claude (Stage: Q = 0.57, *p* = 0.75; Grade: Q = 0.75, *p* = 0.69). In contrast, Gemini showed evidence of session-to-session variation, most clearly for Stage (Q = 10.8, nominal *p* = 0.004); nominal *p* values were 0.020 for Grade (Q = 7.8) and 0.042 for Extent (Q = 6.3), in keeping with its lower Fleiss’ kappa values. Claude’s lower concordance therefore reflected mainly errors that recurred across sessions, whereas Gemini’s discordances were driven by session variability.

Direction and magnitude of Stage errors: Contrary to our expectation that disagreements would be confined to the Stage III/IV boundary, most Stage discordances lay at the lower boundary of Stage III. Across all 945 sessions, Claude produced 42 Stage-discordant responses (35 Stage II and 3 Stage I for Stage III cases; 4 Stage III for Stage IV cases) and Gemini 21 (11 Stage I and 6 Stage II for Stage III cases; 3 Stage III for Stage IV cases; 1 Stage IV for a Stage III case); ChatGPT produced 3 (all Stage III for a single Stage IV case). Fourteen responses in 8 cases (Claude 3, Gemini 11) were two categories below the reference, and 4 of these became the modal decision (Table 3). All Grade discordances at the modal level were between adjacent grades (Claude 2/2 A–B; Gemini one A–B and one B–C); at the session level, one ChatGPT response assigned Grade A to a Grade C case, but this did not affect its modal decision.

The 14 Stage III cases understaged by Claude and/or Gemini shared a distinctive profile: maximal interdental CAL of 5–6 mm (i.e., at or just above the ≥5 mm Stage III threshold), maximal RBL of 7.5–31.5% of root length (i.e., within the coronal third, corresponding to Stage I–II severity), and no tooth loss attributable to periodontitis. By comparison, the 57 Stage III cases that no model understaged had a mean maximal CAL of 6.7 mm and a mean RBL of 45%. Gemini’s Stage I outputs corresponded to cases with RBL below 15%. The experts, following the framework’s instruction that CAL is the primary determinant of severity, assigned Stage III; the pattern is consistent with the two models giving greater weight to the radiographic criterion when the two severity indicators pointed to different Stages. Eleven of these 14 cases were from Center B (Section 3.9).

Sensitivity to the reference standard: When the 7 cases adjudicated by Expert C from summaries only were excluded (*n* = 98), Stage κ was 0.97 (ChatGPT), 0.69 (Claude), and 0.90 (Gemini); Grade κw was 1.00, 0.97, and 0.97; Extent κ was 1.00, 1.00, and 0.92; and full concordance was 97/98, 83/98, and 93/98, respectively. All three models agreed with the adjudicated reference in all 7 of these cases (which were all assigned Stage IV). Restricting the analysis to the 93 cases on which Experts A and B agreed in Round 1 gave Stage κ of 0.97, 0.69, and 0.92, respectively. Among the 12 cases discordant at Round 1, full concordance was 12/12 (ChatGPT), 11/12 (Claude), and 11/12 (Gemini), indicating that LLM disagreement was not concentrated among cases that were also difficult for the experts.

When each expert’s independent Round 1 classification was used directly as the reference instead of the adjudicated consensus (Table 8), Stage κ was 0.87/0.89 (ChatGPT vs. Expert A/B), 0.63/0.65 (Claude), and 0.81/0.83 (Gemini), and full concordance 92–94%, 80–81%, and 90–91%, respectively, compared with a Round 1 inter-expert Stage κ of 0.79 and full agreement of 88.6%. These values arise from different comparison structures (model versus a single rater, and rater versus rater) and are presented for descriptive context only; no formal equivalence or non-inferiority comparison was performed.

### 3.9. Exploratory Mixed-Effects Analysis of Model, Stage, and Center Effects

Reference Stage distribution differed between centers: Center A (Batman) contributed 28 Stage III and 22 Stage IV cases (44% Stage IV), Center B (Burdur) 43 Stage III and 12 Stage IV (22% Stage IV). Modal Stage concordance was 100% for all models in Stage IV cases at Center B and 95.5% at Center A (the single ChatGPT discordance); among Stage III cases it was 100%/100% (ChatGPT), 89.3%/74.4% (Claude), and 100%/93.0% (Gemini) at Centers A/B. The between-center difference was therefore confined to Claude’s and Gemini’s performance in Stage III cases, 11 of the 14 understaged cases (Section 3.8) being from Center B.

In the Bayesian mixed-effects model (all 945 sessions; 0 divergent transitions, all R-hat = 1.00, bulk ESS > 2500), odds of session-level Stage concordance were substantially lower for Claude (OR = 0.02, 95% CrI 0.00–0.06) and Gemini (OR = 0.07, 95% CrI 0.01–0.24) than for ChatGPT, and higher for Stage IV than Stage III cases (OR = 9.9, 95% CrI 1.3–95). After adjustment for model and Stage, the center effect was small and imprecise (Center A vs. Center B: OR = 1.6, 95% CrI 0.3–10). The patient random-intercept standard deviation was large (3.2 on the logit scale), indicating that concordance depended strongly on case-level characteristics. When the Stage term was omitted, the center OR increased to 2.5 (95% CrI 0.5–14), and a Stage × center interaction model gave an interaction OR of 11.7 with a very wide CrI (0.4–445); with only two centers and a Stage distribution that differed between them, the data cannot separate a center effect from the Stage (and case-mix) effect. These ORs are exploratory (Section 2.9).

## 4. Discussion

In this multicenter study of 105 real patients with Stage III–IV periodontitis, three general-purpose LLMs, given standardized summaries and an explicit 2018 AAP/EFP instruction, showed substantial to almost perfect concordance with an adjudicated expert consensus for Stage and Grade, and almost perfect concordance for Extent. The task the models performed should be stated precisely: they applied the classification rules to variables that clinicians had already extracted, coded, and, for Grade modifiers and complexity factors, pre-discretized. They did not read charts or radiographs, and the results should not be read as evidence that these models can diagnose or stage periodontitis from primary clinical data. Concordance was not uniform: ChatGPT reproduced the reference almost exactly, Gemini did so with notable session-to-session variation, and Claude’s Stage concordance (κ = 0.73), though substantial, remained below the other models and reflected a systematic pattern of understaging at the modal level.

Our results are notably higher than those of Tastan Eroglu et al. [13], who found only fair-to-poor agreement (kappa approximately 0.20–0.30) when ChatGPT classified Stage and Grade in 200 patients. The studies differed in at least two important respects. Here, the model received a structured case summary together with the relevant 2018 AAP/EFP criteria; previous work suggests that such domain-specific instructions can materially change classification performance [14]. We also tested newer model generations, and improved agreement has been reported with GPT-5 [15]. Because prompt design and model generation changed at the same time, the present data cannot separate their contributions. They do, however, show that agreement depends strongly on the information and instructions supplied and on the particular model tested.

Grade was more reproducible than Stage, and two explanations should be considered. The first is that Grade is intrinsically easier for these models. The second, which we regard as at least as plausible, is that the task was simplified by the format of the input: Grade modifiers (smoking and HbA1c categories) and the bone loss/age ratio were supplied as discrete values, so that Grade assignment required essentially a look-up, whereas Stage required several findings to be weighed against a compound rule. The same applies to Stage IV determination, for which the complexity factors were supplied as binary flags. The present design cannot distinguish model capability from task simplification, and performance would very likely be lower if the models were given unstructured chart text or uninterpreted radiographic images, from which the relevant variables would first have to be extracted.

The error analysis makes this concrete. Almost all Stage discordances by Claude and Gemini were Stage III cases assigned Stage II or, for Gemini, Stage I, and all shared a profile in which interdental CAL (5–6 mm) met the Stage III threshold while RBL (<33%) did not. The 2018 framework specifies CAL as the primary determinant of severity, with RBL used when CAL is unavailable [2]; the experts applied this hierarchy, whereas the pattern is consistent with the two models giving greater weight to the radiographic percentage when the two indicators conflicted; the models’ internal reasoning was not observed directly. This is an error of rule integration rather than of data extraction, it occurred despite explicit instruction to follow the framework, and in 7 of Claude’s cases it recurred in all three sessions. It also illustrates why the direction of error matters clinically: although one session-level Gemini response overstaged a Stage III case, every classifiable modal Stage discordance in this study understated severity, and a Stage III case labeled Stage I or II would, under the EFP S3 guidelines, be assigned to a less intensive treatment pathway [3,4]. That the cases concerned were borderline on one criterion does not diminish this concern, because such conflicts between clinical and radiographic severity are common in practice.

Repeated testing added information that a single session could not provide, but in two different ways. ChatGPT responded almost identically each time, while Gemini’s lower Fleiss’ kappa values and Cochran’s Q results showed meaningful session-to-session variation. For Gemini, the modal decision rule materially improved apparent performance (full concordance 95.2% for the modal decision versus 85.7–99.0% in single sessions), so that a user relying on one query could obtain a Stage κ of 0.81 rather than 0.92. For Claude, single-session and modal results were similar because its errors were mostly stable. The vote pattern across sessions proved informative: for Grade and Extent, every discordance occurred in a case where the three sessions disagreed, and none where they agreed, and for Stage a split vote raised the probability of error several-fold. In the absence of access to output probabilities, which consumer interfaces do not expose, disagreement between repeated queries is a simple, model-agnostic indicator that a case merits expert review; it cannot, however, flag errors that a model makes consistently. Similar variability across repeated clinical queries has been reported elsewhere [16,17].

The human reference was not itself free of disagreement. Stage agreement between the two experts in Round 1 was κ = 0.79. This figure provides context but is not a direct benchmark for the model results: the adjudicated consensus against which the models were scored was formed from those same experts’ decisions and therefore represents a partly de-noised reference, and the models were given pre-extracted variables. When each expert’s independent classification was used as the reference instead (Table 8), model–expert Stage agreement was 0.87–0.89 for ChatGPT, 0.81–0.83 for Gemini, and 0.63–0.65 for Claude, compared with an inter-expert value of 0.79. These descriptive values provide context but should not be interpreted as demonstrating equivalence, superiority, or non-inferiority to expert performance. International studies have likewise reported imperfect agreement among trained examiners using the same framework [5,6].

This study was not designed to explain why the three models differed. Their architectures, internal instructions, and generation settings are not visible to users and could not be measured directly. The between-model comparisons were exploratory and unadjusted for multiplicity, and because ChatGPT produced only three discordant sessions, the corresponding odds ratios are imprecise. The apparent difference between centers was confined to Stage III cases and coincided with a higher concentration of borderline Stage III cases (low RBL, CAL at threshold) at Center B; once model and Stage were accounted for, the center effect was small and its credible interval wide, and with two centers the data cannot separate center from case mix. If a center effect persists in other datasets, classification support may not perform uniformly across clinical settings, and local validation would be necessary before deployment.

A few directions could extend this work. Testing through API access, rather than the consumer interfaces used here, would allow inference parameters and a pinned model version to be fixed and reported, and would give access to output token probabilities, from which calibration and receiver-operating-characteristic analyses could be derived and low-confidence cases flagged more directly than by repeated querying. A prospective design, in which the clinician-prepared summary is generated as part of routine care rather than drawn from archived records, would show how these tools perform once built into clinical workflow. Pairing LLM-based staging and grading with automated extraction of the underlying clinical and radiographic variables would also move the evaluation closer to the full diagnostic task, rather than rule application to an already-summarized case. Deliberately enriching a test set with cases in which clinical and radiographic severity conflict would test the rule-integration failure identified here.

Previous periodontal studies describe moderate-to-good performance on knowledge questions [7,8,11,12], variable treatment-planning quality [9], and errors in guideline extraction [10]. Read alongside our findings, this literature suggests that performance is closely tied to the task, the prompt, and the context provided, and that a carefully specified prompt may be particularly useful in dental education for staging and grading exercises. Gemini’s greater session-to-session variability also argues against relying on a single response when reproducibility matters: a clinician who queried the same case twice could receive a different Stage or Grade.

Because these tools can err, and because their errors here were systematically in the direction of understating disease, the question of who bears responsibility for a classification error deserves explicit comment. None of the evaluated platforms is authorized as a medical device for periodontal diagnosis, and their terms of use disclaim clinical reliability. The allocation of legal responsibility for errors involving AI-assisted clinical decisions remains jurisdiction- and context-dependent and may involve clinicians, healthcare institutions, developers, or manufacturers, depending on the regulatory status and intended use of the system and on the applicable standard of care [26,27]; legal commentary indicates that a clinician who relies on an erroneous output that departs from the standard of care is unlikely to be shielded by the tool’s involvement, whereas the liability of the provider of a general-purpose model used outside its intended purpose is largely untested [26,27]. In the workflow evaluated here, the LLM output does not replace professional judgment: the treating clinician selects and verifies the input data, is trained to recognize an implausible output, and remains responsible for the classification used in patient care. Accordingly, our findings should not be interpreted as transferring diagnostic responsibility from the clinician to the model, and this is a further reason why we regard the evaluated use as appropriate only under specialist supervision.

### Limitations

This study has several limitations. The LLMs did not review the raw charts or radiographs available to the experts. Instead, they received clinician-prepared summaries in which complexity factors, Grade modifiers, and tooth counts for Extent had already been extracted and coded. We therefore evaluated application of classification rules to prepared variables, not image interpretation or the full diagnostic process, and the design cannot separate model capability from the simplification of the task that pre-structuring the input entails. The results do not show that these models can obtain the same variables reliably from charts, radiographs, or free text, and we could not examine how they internally applied the 30% threshold for Extent. Performance may also differ with a less structured prompt. Second, for the 7 cases adjudicated by Expert C, the reference was formed from the standardized summary rather than the complete record, an inconsistency in the reference standard; sensitivity analyses excluding these cases, and using each Round 1 expert as the reference, did not change the conclusions. Third, several subgroups were small: Grade A (*n* = 7), localized Extent (*n* = 16), and Stage IV (*n* = 34). Kappa estimates involving these categories have wide intervals, weighted kappa is sensitive to marginal imbalance, and conclusions about the direction of Grade errors rest on only four modal discordances. Fourth, the model labels reported are the consumer-facing labels displayed during 18–24 July 2026 (ChatGPT GPT-5.6 Sol, Claude claude-sonnet-5-medium, Gemini 3.1 Pro). Vendors may modify the underlying model or serving configuration without changing the displayed label [28], so the absence of label drift does not establish the absence of model drift, and the results cannot be attributed to a verifiable model version; API access with a pinned version would be required for that. Fifth, the sample size was planned for the precision of kappa, not for between-model comparisons, which are exploratory and unadjusted for multiplicity; the mixed-effects estimates for ChatGPT, with three discordant sessions, are particularly imprecise. Sixth, the modal decision rule used for the primary analysis represents a best-case use pattern; single-session results (Table 7) are the appropriate reference for ordinary single-query use. Because Stage I and II cases were excluded, the findings may be affected by spectrum restriction and do not extend to earlier disease; in particular, we cannot tell whether the understaging tendency observed at the lower boundary of Stage III would also appear as overstaging of Stage II cases. Both centers were in Türkiye, so geographic representativeness remains limited. We included only complete cases and cannot infer how the models would respond to missing information. The mixed-effects, error-pattern, and Cochran’s Q analyses were not the basis of the sample-size calculation and should be regarded as hypothesis-generating.

These considerations, taken as a whole, point to a clear intended use for the evaluated workflow. Because the LLMs applied classification rules to information a clinician had already extracted and structured, rather than demonstrating independent diagnostic reasoning, we regard exploratory use as appropriate only for clinician-supervised classification checking or dental education, in which a periodontally trained clinician prepares the summary, checks it against the complete record, and reviews the output, with particular attention to cases in which clinical and radiographic severity disagree. The findings do not support autonomous use, use without specialist oversight, or extension to unstructured or unsupervised settings.

## 5. Conclusions

When given clinician-prepared, standardized case summaries and explicit 2018 AAP/EFP instructions, ChatGPT, Claude, and Gemini applied the classification rules with substantial to almost perfect concordance with an adjudicated expert consensus for Stage, Grade, and Extent. This performance reflects rule application to pre-extracted variables, not independent periodontal diagnosis, and part of the high Grade concordance is likely attributable to the pre-coded format of the inputs. Performance was model-specific: ChatGPT was almost perfectly concordant and reproducible; Gemini showed greater session-to-session variability, particularly for Stage, so that single-query performance was lower than the modal result; and Claude, together with Gemini, systematically understaged Stage III cases in which radiographic bone loss was mild relative to clinical attachment loss. Disagreement between repeated queries identified most Grade and Extent errors but not consistent Stage errors. These findings support a role for general-purpose LLMs only as clinician-supervised classification-checking and educational tools, with the treating clinician remaining responsible for the diagnosis, and they warrant local validation and version-controlled testing before any further application.

## Figures and Tables

**Table 1:** Patient and case characteristics of the analyzed sample (*N* = 105).

Characteristic	Value	*n* (%)/Range
Age, years (mean ± SD)	56.8 ± 11.9	range 29–84
Sex: male/female	61/44	58.1%/41.9%
Center: A (Batman)/B (Burdur)	50/55	47.6%/52.4%
Smoking: current/non-smoker/former	46/58/1	43.8%/55.2%/1.0%
Diabetes: present/absent	10/95	9.5%/90.5%
Reference Stage: III/IV	71/34	67.6%/32.4%
Reference Grade: A/B/C	7/43/55	6.7%/41.0%/52.4%
Reference Extent: localized/generalized	16/89	15.2%/84.8%

SD, standard deviation.

**Table 2:** Model version and reproducibility (Fleiss’ kappa across three sessions).

Model	Platform	Platform-Displayed Model Label	Testing Period	Fleiss κ (Stage/Grade/Extent)
ChatGPT	chatgpt.com	GPT-5.6 Sol	18–24 July 2026	1.00/0.99/1.00
Claude	claude.ai	claude-sonnet-5-medium	18–24 July 2026	0.85/0.91/1.00
Gemini	gemini.com	Gemini 3.1 Pro	18–24 July 2026	0.81/0.89/0.85

κ, Fleiss’ kappa; Fleiss’ kappa per Landis & Koch [23]; no change in the displayed label during testing (Section 3.3); labels are as displayed in the consumer interface and do not identify a verifiable backend model version (Section Limitations).

**Table 3:** Stage concordance (Cohen’s kappa; α = 0.025, 97.5% bootstrap CI).

Model	*N*	Observed Agreement	κ	97.5% CI
ChatGPT	105	99.0%	0.98	0.91–1.00
Claude	105	85.7%	0.73	0.59–0.86
Gemini	105	96.2%	0.92	0.82–1.00

*N*, number of cases; CI, confidence interval; κ, Cohen’s kappa. Bootstrap CIs (Section 2.9), 97.5% level.

**Table 4:** Grade concordance (linear-weighted kappa, A/B/C; α = 0.025, 97.5% bootstrap CI).

Model	*N*	Weighted Po	κw	97.5% CI
ChatGPT	105	100%	1.00	1.00–1.00
Claude	105	99.0%	0.97	0.91–1.00
Gemini	105	99.0%	0.97	0.91–1.00

*N*, number of cases; Po, weighted observed agreement; κw, linearly weighted kappa; CI, confidence interval. Bootstrap CIs (Section 2.9), 97.5% level; discordance direction is described in Section 3.5.

**Table 5:** Extent and full concordance (Cohen’s kappa, binary; α = 0.05).

Model	Extent κ (95% CI)	Full Concordance, *n/N* (%)	95% CI
ChatGPT	1.00 (1.00–1.00)	104/105 (99.0%)	97–100%
Claude	1.00 (1.00–1.00)	90/105 (85.7%)	79–92%
Gemini	0.93 (0.80–1.00)	100/105 (95.2%)	91–99%

κ, Cohen’s kappa; CI, confidence interval; *n/N*, number concordant/total number evaluated. Bootstrap CIs (Section 2.9), 95% level.

**Table 6:** Vote pattern across the three sessions and modal Stage concordance (*N* = 105 cases per model).

Model	3/3 Concordant	3/3 Discordant	2/1 Concordant	2/1 Discordant	1/1/1	Discordance, Split vs. 3/3
ChatGPT	104	1	0	0	0	— vs. 1.0%
Claude	85	7	5	7	1	58% vs. 7.6%
Gemini	89	1	12	3	0	20% vs. 1.1%

Grade: Claude 97 unanimous (0 discordant) and 8 split (2 discordant); Gemini 95 unanimous (0 discordant) and 10 split (2 discordant); ChatGPT 104 unanimous and 1 split (concordant). Extent: Gemini 99 unanimous (0 discordant) and 6 split (2 discordant); ChatGPT and Claude 105 unanimous. —, not applicable (ChatGPT produced no split Stage decisions).

**Table 7:** Single-session concordance with the adjudicated reference (each of the three sessions analyzed separately; *N* = 105 per session).

Model	Session	Stage κ (97.5% CI)	Grade κw (97.5% CI)	Extent κ (95% CI)	Full Concordance, *n/N* (%)
ChatGPT	1	0.98 (0.91–1.00)	0.97 (0.89–1.00)	1.00 (1.00–1.00)	103/105 (98.1)
ChatGPT	2	0.98 (0.92–1.00)	1.00 (1.00–1.00)	1.00 (1.00–1.00)	104/105 (99.0)
ChatGPT	3	0.98 (0.91–1.00)	1.00 (1.00–1.00)	1.00 (1.00–1.00)	104/105 (99.0)
Claude	1	0.73 (0.59–0.85)	0.95 (0.88–1.00)	1.00 (1.00–1.00)	89/105 (84.8)
Claude	2	0.74 (0.60–0.87)	0.94 (0.86–1.00)	1.00 (1.00–1.00)	90/105 (85.7)
Claude	3	0.75 (0.61–0.88)	0.95 (0.89–1.00)	1.00 (1.00–1.00)	91/105 (86.7)
Gemini	1	0.81 (0.67–0.92)	0.90 (0.80–0.97)	0.81 (0.61–0.96)	90/105 (85.7)
Gemini	2	0.98 (0.91–1.00)	1.00 (1.00–1.00)	1.00 (1.00–1.00)	104/105 (99.0)
Gemini	3	0.81 (0.68–0.92)	0.93 (0.84–0.99)	0.89 (0.74–1.00)	92/105 (87.6)

κ, Cohen’s kappa; κw, linearly weighted kappa; CI, bootstrap confidence interval (10,000 patient-level resamples). Modal-decision results are in Table 3, Table 4 and Table 5.

**Table 8:** Modal LLM concordance with each expert’s independent Round 1 classification and with the adjudicated consensus (*N* = 105).

Comparison	Stage κ (97.5% CI)	Grade κw (97.5% CI)	Extent κ	Full Agreement, %
ChatGPT vs. adjudicated consensus	0.98 (0.91–1.00)	1.00	1.00	99.0
ChatGPT vs. Expert A	0.87 (0.73–0.98)	0.96 (0.89–1.00)	1.00	92.4
ChatGPT vs. Expert B	0.89 (0.76–0.98)	0.96 (0.89–1.00)	1.00	94.3
Claude vs. adjudicated consensus	0.73 (0.59–0.86)	0.97 (0.91–1.00)	1.00	85.7
Claude vs. Expert A	0.63 (0.48–0.77)	0.92 (0.85–0.98)	1.00	80.0
Claude vs. Expert B	0.65 (0.50–0.79)	0.92 (0.84–0.98)	1.00	81.0
Gemini vs. adjudicated consensus	0.92 (0.82–1.00)	0.97 (0.91–1.00)	0.93	95.2
Gemini vs. Expert A	0.81 (0.66–0.93)	0.96 (0.89–1.00)	0.93	89.5
Gemini vs. Expert B	0.83 (0.69–0.94)	0.93 (0.84–0.99)	0.93	90.5
Expert A vs. Expert B (Round 1)	0.79 (0.62–0.93)	0.91 (0.82–0.98)	1.00	88.6

κ, Cohen’s kappa; κw, linearly weighted kappa; CI, bootstrap confidence interval. Expert A and B classifications are their independent Round 1 decisions before consensus or adjudication.

## Data Availability

The de-identified case-level dataset, the statistical analysis workbook, and the analysis code/scripts (Python) used to compute kappa statistics, bootstrap confidence intervals, and the Bayesian mixed-effects model (Section 2.9) supporting the findings of this study are available from the corresponding author upon reasonable request, subject to institutional data-sharing policies and the retrospective ethics approval under which patient data were collected.

## Supplementary Materials

The following supporting information can be downloaded at https://www.mdpi.com/article/10.3390/jcm15197585/s1: File S1: STROBE Checklist (Cohort Studies); File S2: GRRAS Checklist (Guidelines for Reporting Reliability and Agreement Studies); File S3: STARD-AI Checklist (incorporating STARD 2015); File S4: Exact System Instruction and Standardized Case-Form Template Used with the LLMs (including an illustrative model-output format, the full sample-size pilot rule, and the specification and code of the Bayesian mixed-effects model); File S5: TRIPOD-LLM Checklist.

## Abbreviations

The following abbreviations are used in this manuscript:

LLMs	Large Language Models
AAP	American Academy of Periodontology
EFP	European Federation of Periodontology
CIs	Confidence intervals
CAL	Clinical attachment loss
RBL	Radiographic bone loss
OR	Odds ratio
CrI	Credible interval
