# Supplementary material for: Staging and Grading Advanced Periodontitis with Large Language Models from Standardized Case Summaries: A Multicenter Concordance and Reproducibility Study
Source: J Clin Med. 2026 Sep 30;15(19):7585. doi: 10.3390/jcm15197585 (PMC13665847; doi:10.3390/jcm15197585)
Supplement: Supplementary file 1 [file jcm-15-07585-s001.zip › File_S1_STROBE_Checklist_R1.pdf]

SUPPLEMENTARY FILE S1 — STROBE Checklist (Cohort Studies)

Manuscript: Staging and Grading Advanced Periodontitis with Large Language Models from Standardized Case Summaries: A Multicenter Concordance and Reproducibility Study

Authors: Mehmet Kızıltoprak, Mustafa Karaca, Mustafa Özay Uslu

This checklist follows the STROBE Statement (von Elm E, Altman DG, Egger M, Pocock SJ, Gøtzsche PC, Vandenbroucke JP. The Strengthening the Reporting of Observational Studies in Epidemiology (STROBE) statement: guidelines for reporting observational studies. Lancet. 2007;370(9596):1453–1457), used here to report the underlying multicentre, retrospective observational design of this concordance study. STROBE items relating to the AI index tests and to inter-rater reliability are cross-referenced to Supplementary Files 2 (GRRAS) and 3 (STARD-AI), which provide the AI-specific and reliability-specific reporting detail.

Section/Topic	No.	Item	Reported in manuscript
Title and abstract	1a	Indicate the study's design with a commonly used term in the title or the abstract	*Title ("Multicentre Retrospective Study")*
	1b	Provide in the abstract an informative and balanced summary of what was done and what was found	*Abstract*
Introduction	2	Explain the scientific background and rationale for the investigation being reported	*Introduction*
	3	State specific objectives, including any prespecified hypotheses	*Introduction, final paragraph*
Methods	4	Present key elements of study design early in the paper	*Section 2.1*
	5	Describe the setting, locations, and relevant dates, including periods of recruitment, exposure, follow-up, and data collection	*Sections 2.1–2.2 (centres; record collection 8–18 Jul 2026, following 7 Jul 2026 ethics approval), 2.4 (testing period 18–24 Jul 2026)*
	6a	Give the eligibility criteria, and the sources and methods of selection of participants	*Section 2.2*
	6b	For matched studies, give matching criteria and number of exposed/unexposed (not applicable — not a matched design)	*Not applicable*
	7	Clearly define all outcomes, exposures, predictors, potential confounders, and effect modifiers; give diagnostic criteria if applicable	*Sections 2.6–2.7 (Section 3 dimensions and 2018 AAP/EFP criteria)*
	8*	For each variable of interest, give sources of data and details of methods of assessment; describe comparability of assessment methods across groups	*Sections 2.3 (expert assessment), 2.4 (LLM assessment), 2.6 (radiographic measurement, two independent examiners)*
	9	Describe any efforts to address potential sources of bias	*Sections 2.5 (blinding), 2.6 (asymmetry acknowledged), 4.1 (limitations)*
	10	Explain how the study size was arrived at	*Section 2.8*
	11	Explain how quantitative variables were handled in the analyses; describe groupings if applicable	*Section 2.9 (kappa/ICC computation); Extent 30% threshold, Section 2.6*

Section/Topic	No.	Item	Reported in manuscript
	12a–e	Describe all statistical methods; subgroup/interaction analyses; handling of missing data; sensitivity analyses	*Section 2.9*
Results	13a–c	Numbers of individuals at each stage of study; reasons for non-participation; consider a flow diagram	*Section 3.1; Fig. 1*
	14a–c	Characteristics of participants and information on exposures/confounders; missing data; follow-up time	*Section 3.1; Table 1*
	15*	Report numbers of outcome events or summary measures over time	*Sections 3.4–3.8; Tables 2–5*
	16a–c	Unadjusted/adjusted estimates and precision (e.g., 95% CI); category boundaries for categorized continuous variables	*Section 2.6 (30% Extent threshold); Tables 3–5 (kappa with 95% CI)*
	17	Report other analyses done, e.g., subgroup and sensitivity analyses	Sections 3.7–3.9 (single-session analysis, Table 7; vote-pattern analysis, Table 6; sensitivity analyses excluding the 7 summary-adjudicated cases and using each expert as reference, Table 8; error-direction analysis; Cochran's Q; Bayesian mixed-effects model)
Discussion	18	Summarise key results with reference to study objectives	*Discussion, opening paragraph*
	19	Discuss limitations, taking into account sources of potential bias or imprecision; direction and magnitude of bias	*Section 4.1*
	20	Give a cautious overall interpretation considering objectives, limitations, similar studies, other evidence	*Discussion*
	21	Discuss the generalisability (external validity) of the study results	*Section 4.1*
Other information	22	Give the source of funding and the role of the funders	*Funding statement*

** Item 8 and 15 should be given separately for exposed and unexposed groups per the original STROBE wording; in this concordance design, the equivalent comparison is between models/dimensions, reported in Tables 2–5. Page numbers refer to the main manuscript (Word/PDF pagination) and may shift slightly with journal typesetting; section numbers are stable and should be used as the primary cross-reference.*
