# Supplementary material for: Staging and Grading Advanced Periodontitis with Large Language Models from Standardized Case Summaries: A Multicenter Concordance and Reproducibility Study
Source: J Clin Med. 2026 Sep 30;15(19):7585. doi: 10.3390/jcm15197585 (PMC13665847; doi:10.3390/jcm15197585)
Supplement: Supplementary file 1 [file jcm-15-07585-s001.zip › File_S2_GRRAS_Checklist_R1.pdf]

# SUPPLEMENTARY FILE S2 — GRRAS Checklist (Guidelines for Reporting Reliability and Agreement Studies)

*Manuscript: Staging and Grading Advanced Periodontitis with Large Language Models* from Standardized Case Summaries: A Multicenter Concordance and Reproducibility Study

*Authors: Mehmet Kızıltoprak, Mustafa Karaca, Mustafa Özay Uslu*

This checklist follows GRRAS (Kottner J, Audigé L, Brorson S, Donner A, Gajewski BJ, Hróbjartsson A, et al. Guidelines for Reporting Reliability and Agreement Studies (GRRAS) were proposed. J Clin Epidemiol. 2011;64(1):96–106), used here to report the three reliability/agreement components of this study: (i) Round 1 inter-expert agreement (Cohen's kappa/weighted kappa) prior to adjudication, (ii) inter-examiner reliability of the radiographic bone-loss measurement (ICC), and (iii) inter-session (test–retest) reproducibility of each LLM's classification across three independent sessions (Fleiss' kappa).

| Section/Topic | No. | Item | Reported in manuscript |
|---|---|---|---|
| Title/Abstract | 1 | Identify in title or abstract that interrater/intrarater reliability or agreement was investigated | *Title ("Concordance"), Abstract ("reproducibility (Fleiss' kappa)")* |
| Introduction | 2 | Name and describe the diagnostic or measurement device of interest explicitly | *Sections 2.3 (expert reference standard classification), 2.4 (LLM index tests), 2.6 (radiographic bone-loss measurement)* |
| | 3 | Specify the subject population of interest | *Section 2.2* |
| | 4 | Specify the rater population of interest (if applicable) | *Sections 2.3 (two centre periodontologists ± third adjudicator), 2.4 (three LLMs: ChatGPT, Claude, Gemini), 2.6 (two independent radiographic examiners)* |
| | 5 | Describe what is already known about reliability and agreement and provide a rationale for the study | *Introduction, paragraphs 1–2 (human inter-expert agreement benchmark)* |
| Methods | 6 | Explain how the sample size was chosen; state the determined number of raters, subjects/objects, and replicate observations | *Section 2.8 (n=105 cases; 2 expert raters; 3 models × 3 sessions = 9 replicate LLM observations per case)* |
| | 7 | Describe the sampling method | *Sections 2.2 (retrospective clinic records), 2.8 (pilot-based accrual rule)* |
| | 8 | Describe the measurement/rating process (e.g., time interval between repeated measurements, availability of clinical information, blinding) | *Section 2.4 (≥48 h between LLM sessions, memory disabled), Section 2.5 (blinding)* |
| | 9 | State whether measurements/ratings were conducted independently | *Sections 2.3 (independent Round 1 expert classification), 2.4 (independent LLM sessions)* |
| | 10 | Describe the statistical analysis | *Section 2.9* |
| Results | 11 | State the actual number of raters and subjects/objects included and the number of replicate observations conducted | *Section 3.1 (n=105 cases), Section 3.3 (945 LLM sessions = 105 × 3 models × 3 sessions)* |
| | 12 | Describe the sample characteristics of raters and subjects (e.g., training, experience) | *Table 1 (patient/case characteristics); expert raters are centre-based periodontologists (Section 2.3); both board-* |

| Section/Topic | No. | Item | Reported in manuscript |
| --- | --- | --- | --- |
| | | | *certified, with 7 and 8 years of post-certification clinical experience, respectively; a third, independent periodontologist (Expert C) with 12 years of post-doctoral experience adjudicated the 7 discordant cases not resolved by structured consensus (Section 2.3)* |
| | 13 | Report estimates of reliability and agreement including measures of statistical uncertainty | *Section 3.2 (inter-expert κ, radiographic ICC);* Section 3.7 and Table 2 (Fleiss' κ); single-session concordance, Table 7; vote-pattern analysis, Table 6 |
| Discussion | 14 | Discuss the practical relevance of results | *Discussion* |
| Auxiliary material | 15 | Provide detailed results if possible (e.g., online) | *Data Availability Statement — de-identified case-level dataset, analysis workbook, and analysis code/scripts available from the corresponding author on reasonable request* |

*Page numbers refer to the main manuscript (Word/PDF pagination) and may shift slightly with journal typesetting; section numbers are stable and should be used as the primary cross-reference.*
