# Supplementary material for: Staging and Grading Advanced Periodontitis with Large Language Models from Standardized Case Summaries: A Multicenter Concordance and Reproducibility Study
Source: J Clin Med. 2026 Sep 30;15(19):7585. doi: 10.3390/jcm15197585 (PMC13665847; doi:10.3390/jcm15197585)
Supplement: Supplementary file 1 [file jcm-15-07585-s001.zip › File_S3_STARDAI_Checklist_R1.pdf]

SUPPLEMENTARY FILE S3 — STARD-AI Checklist (incorporating STARD 2015)

Manuscript: Staging and Grading Advanced Periodontitis with Large Language Models from Standardized Case Summaries: A Multicenter Concordance and Reproducibility Study

Authors: Mehmet Kızıltoprak, Mustafa Karaca, Mustafa Özay Uslu

This checklist follows STARD-AI (Sounderajah V, Guni A, Liu X, et al. The STARD-AI reporting guideline for diagnostic accuracy studies using artificial intelligence. Nat Med. 2025;31(10):3283–3289), the AI-specific extension of STARD 2015 (Bossuyt PM, Reitsma JB, Bruns DE, et al. STARD 2015: an updated list of essential items for reporting diagnostic accuracy studies. BMJ. 2015;351:h5527). The 40-item STARD-AI checklist supersedes and incorporates all STARD 2015 items (4 modified: items 1, 3, 7, 25; 14 new items marked * below), so a single combined checklist is presented rather than two overlapping tables. STARD-AI explicitly supports its use for studies assessing the diagnostic/classification accuracy of large language models, which is the design used here.

Terminology note: per the pre-specified terminology commitment of this study (Section 2.1), the manuscript reports concordance/agreement (kappa) rather than sensitivity/specificity/accuracy. Where the STARD-AI item wording refers to "diagnostic accuracy", this checklist maps it to the equivalent concordance measure reported in the manuscript; STARD-AI itself notes that its items remain applicable to studies of LLM diagnostic/classification output even when framed in agreement rather than accuracy terms.

Section/Topic	No.	Item	Reported in manuscript
Title or abstract	1†	Identification as a study reporting AI-centred diagnostic accuracy and reporting at least one measure of accuracy within title or abstract	*Title/Abstract report concordance (Cohen's/weighted kappa) rather than sensitivity/specificity, consistent with the pre-specified concordance terminology (Section 2.1); kappa serves as the equivalent agreement/accuracy measure*
Abstract	2	Structured summary of study design, methods, results and conclusions	*Abstract (Objectives/Materials and Methods/Results/Conclusions/Clinical Relevance)*
Introduction	3†	Scientific and clinical background, including intended use of the index test, whether novel/established, and workflow integration	*Introduction*
	4	Study objectives and hypotheses	*Introduction, final paragraph*
Methods — Study design	5	Whether data collection was planned before the index test and reference standard were performed (prospective) or after (retrospective)	*Section 2.1 — retrospective record sampling with a prospectively locked analysis protocol*
Methods — Ethics	6*	Formal approval from an ethics committee; if not required, justify why	*Section 2.1; Declarations — Ethics approval*
Methods — Participants	7†	Eligibility criteria: separate inclusion/exclusion criteria, listed in order applied, at both participant and data level	*Section 2.2*
	8	On what basis potentially eligible participants were identified	*Section 2.2*
	9	Where and when potentially eligible participants were identified (setting, location, dates)	*Sections 2.1–2.2 (two centres; patient records collected 8–18 Jul 2026, following 7 Jul 2026 ethics approval), 2.4 (testing period 18–24 Jul 2026)*

Section/Topic	No.	Item	Reported in manuscript
	10	Whether participants formed a consecutive, random or convenience series	*Section 2.2 — consecutive retrospective clinic records meeting eligibility criteria*
Methods — Dataset	11*	Source of the data and whether routinely collected, specifically collected, or from an open-source repository	*Section 2.2 — routinely collected retrospective clinical records from two periodontology clinics*
	12*	Who undertook the annotations for the dataset (experience, background) and how	*Section 2.3 — two centre-based periodontologists (Expert A/B), both board-certified with 7 and 8 years of post-certification clinical experience, respectively, plus a third, independent periodontologist (Expert C, 12 years of post-doctoral experience) who adjudicated the 7 discordant cases not resolved by structured consensus; Section 2.6 — clinician-derived radiographic summary by two independent examiners*
	13*	Devices/software used to capture data; software version used to engineer the index test, highlighting intended use	*Table 2 — model name, platform, and* platform-displayed model label for each LLM (ChatGPT GPT-5.6 Sol/chatgpt.com; Claude claude-sonnet-5-medium/claude.ai; Gemini 3.1 Pro/gemini.com)
	14*	Data acquisition protocols and data preprocessing, in sufficient detail to allow replication	*Sections 2.4 (standardized case form), 2.6 (radiographic summary derivation and 30% Extent threshold)*
Methods — Test methods	15a	Index test, in sufficient detail to allow replication	*Section 2.4*
	15b*	How the index test was developed, including training/validation/testing/external evaluation and sample sizes	*Not applicable — pre-trained, commercially released general-purpose LLMs were evaluated as-is; no model training/fine-tuning was performed by the authors*
	15c	Definition of and rationale for test positivity cutoffs or result categories, distinguishing pre-specified from exploratory	*Sections 2.6 (30% Extent threshold), 2.7 (modal decision rule, unclassifiable rule), all pre-specified*
	15d*	The specified end-user of the index test and the level of expertise required of users	*Section 2.4 — system instruction frames the model as a clinical periodontology assistant intended for clinician use; Discussion, Section 4.1 () explicitly specifies the intended end-user as a periodontology-trained user who reviews LLM output against the complete clinical record, and states that findings do not support use by non-specialist personnel without such review*
	16a	Reference standard, in sufficient detail to allow replication	*Section 2.3*
	16b	Rationale for choosing the reference standard (if alternatives exist)	*Section 2.3*
	16c	Definition of and rationale for test positivity cutoffs/categories of the reference standard, pre-specified vs exploratory	*Sections 2.3, 2.6 (30% Extent threshold applied identically by experts)*
	17a	Whether clinical information and reference standard results were available to the performers/readers of the index test	*Section 2.5 — LLMs never had access to expert/reference output*
	17b	Whether clinical information and index test results were available to the assessors of the reference standard	*Section 2.5 — experts never had access to LLM output*
Methods — Analysis	18	Methods for estimating or comparing measures of diagnostic accuracy	*Section 2.9*

Section/Topic	No.	Item	Reported in manuscript
	19	How indeterminate index test or reference standard results were handled	*Section 2.7 — unclassifiable/no-stable-modal-decision rule*
	20	How missing data on the index test and reference standard were handled	*Sections 2.2 (eligibility criteria; no eligible record was excluded), 2.7 (session/response-level missing-data rules)*
	21	Any analyses of variability in diagnostic accuracy, distinguishing pre-specified from exploratory	*Section 2.9 —* Cochran's Q (exploratory, multiplicity-unadjusted, nominal p values), single-session analysis (Table 7), vote-pattern analysis (Table 6), sensitivity analyses of the reference standard (Table 8), and the Bayesian mixed-effects model (Section 3.9)
	22	Intended sample size and how it was determined	*Section 2.8*
	23*	Details of any performance error analysis and algorithmic bias/fairness assessments, if undertaken	*Section 3.8 — descriptive error-type analysis, extended to adjacent- vs non-adjacent-category severity classification* (Section 3.8: most Stage discordances by Claude and Gemini were Stage III cases assigned Stage II or I, including four modal decisions two categories below the reference; all classifiable modal Stage discordances understated severity; the mechanism—conflict between CAL and radiographic bone loss in borderline Stage III cases—is analysed) and Grade-error-direction (undergrading vs overgrading) analysis; the between-centre difference (Section 3.9; small and imprecise after adjustment for model and Stage) is discussed as a possible equity consideration (Discussion), though a formal demographic subgroup fairness/bias assessment (e.g., by patient age, sex, or comorbidity) was not undertaken and remains noted as a limitation (Section 4.1)
Results — Participants/dataset	24	Flow of participants, using a diagram	*Section 3.1 (135 patients screened; 30 excluded per Section 2.2 criteria; 105 eligible patients included across two centres); Fig. 1*
	25†	Baseline demographic, clinical and technical characteristics of training, validation and test sets, if applicable	*Table 1 — single evaluation set (no training/validation split, as no model training was performed)*
	26a	Distribution of severity of disease in those with the target condition	*Table 1 — Stage III/IV and Grade A/B/C distribution*
	26b	Distribution of alternative diagnoses in those without the target condition	*Not applicable — all included cases had confirmed Stage III/IV periodontitis; no differential-diagnosis comparator group*
	27	Time interval and any clinical interventions between index test and reference standard	*Section 2.4 — LLM summaries and expert classification were both derived from the same underlying clinical record at the time of retrospective data extraction; no intervening clinical intervention*
	28*	Whether the datasets represent the distribution of the target condition expected from the intended use population	*Section 4.1 — achieved Stage/Grade distribution vs a priori balance target discussed as a limitation*
	29*	For external evaluation on an independent dataset, an assessment of how this differs from the	*Not applicable — single-cohort study; no external validation dataset was*

Section/Topic	No.	Item	Reported in manuscript
		training/validation/test sets	*evaluated, noted as a limitation for future work*
Results — Test results	30	Cross-tabulation of the index test results (or their distribution) by the results of the reference standard	*Confusion matrices underlying Tables 3–5 (Stage, Grade, Extent); summarized in Sections 3.4–3.6*
	31	Estimates of diagnostic accuracy and their precision (e.g., 95% CI)	*Tables 2–5*
	32	Any adverse events from performing the index test or the reference standard	*Not applicable — retrospective, non-interventional evaluation using de-identified summaries; no patient-facing procedure was performed*
Discussion	33	Study limitations, including sources of potential bias, statistical uncertainty and generalisability	*Section 4.1*
	34	Implications for practice, including the intended use and clinical role of the index test	*Discussion and Conclusion*
	35*	Ethical considerations and adherence to ethical standards associated with use of the index test and issues of fairness	*Declarations — Ethics approval; Grade error-direction discussion (undergrading vs overgrading) touches on clinical-safety fairness; Discussion ()* additionally discusses the between-centre difference (Section 3.9; not separable from case mix) as a possible equity consideration (uneven performance across clinical settings/case-mix), though a formal demographic fairness assessment was not undertaken (Section 4.1)
Other information	36	Registration number and name of registry	*Not registered in a public study registry; the underlying protocol was reviewed and approved solely by the institutional ethics committee (decision no. GO 2026/3358)*
	37	Where the full study protocol can be accessed	*Declarations — Availability of data and materials: available from the corresponding author on reasonable request*
	38	Sources of funding and other support; role of funders	*Declarations — Funding*
	39*	Commercial interests, if applicable	*Declarations — Competing interests: the three evaluated platforms (OpenAI/ChatGPT, Anthropic/Claude, Google/Gemini) had no role in study design, funding, conduct, or reporting*
	40a*	Availability of datasets and code, detailing any restrictions on reuse and repurposing	*Declarations — Availability of data and materials / Code availability*
	40b*	Whether outputs are stored, auditable and available for evaluation, if necessary	*Section 2.4, and Table 2 —* every LLM session's output, displayed model label, platform, and testing window were logged in the study workbook and are retained for audit

*† Modified from STARD 2015. * New item introduced by STARD-AI (not present in STARD 2015). Page numbers refer to the main manuscript (Word/PDF pagination) and may shift slightly with journal typesetting; section numbers are stable and should be used as the primary cross-reference.*
