# Supplementary material for: Staging and Grading Advanced Periodontitis with Large Language Models from Standardized Case Summaries: A Multicenter Concordance and Reproducibility Study
Source: J Clin Med. 2026 Sep 30;15(19):7585. doi: 10.3390/jcm15197585 (PMC13665847; doi:10.3390/jcm15197585)
Supplement: Supplementary file 1 [file jcm-15-07585-s001.zip › File_S4_Exact_LLM_Prompt_R1.pdf]

## SUPPLEMENTARY FILE S4 — Exact System Instruction and Standardized Case-Form Template Used with the LLMs

*Manuscript: Staging and Grading Advanced Periodontitis with Large Language Models* from Standardized Case Summaries: A Multicenter Concordance and Reproducibility Study

*Authors: Mehmet Kızıltoprak, Mustafa Karaca, Mustafa Özay Uslu*

This file reproduces, verbatim and unmodified, the fixed system instruction and the standardized case-form template that were presented to ChatGPT, Claude, and Gemini for every case and every session (Section 2.4 of the main manuscript). The template's bracketed fields (e.g., "[___]") were populated with case-specific, de-identified values drawn from the standardized clinical summary before each session; no other wording was added, removed, or reordered across cases, models, or sessions. This exact wording is provided to support independent replication.

### A. Fixed System Instruction (identical for ChatGPT, Claude, and Gemini; unmodified across all cases and sessions)

```
You are a clinical periodontology assistant.

Answer the question based solely on the patient data provided.

Do not request additional information.

Do not invent information that is not present in the case summary.

Base your answer strictly on the 2018 AAP/EFP classification of periodontal
diseases
(Tonetti et al. 2018, J Clin Periodontol 45 Suppl 20: S149-S161).

For Stage IV classification, at least ONE of the following two criteria is
sufficient:

(a) at least one complexity factor is present (posterior bite support loss,
pathological
migration, or masticatory dysfunction), OR

(b) tooth loss due to periodontitis is >=5, even if none of the complexity
factors in (a)
are present. Use the "Tooth loss due to periodontitis" value given below for
criterion (b).

Provide:

(1) Final decision - one sentence.

(2) Rationale - maximum two sentences.
```

*Note: this system instruction deliberately does not mention "Extent" — the model was required to compute the localized/generalized distinction itself from the affected-tooth and total-remaining-tooth counts given in the case form below (Section 2.6 of the main manuscript).*

### B. Standardized Case Form Template (English; bracketed fields populated per case, all other wording unmodified)

```
--- PERIODONTAL PATIENT CASE ---


DEMOGRAPHIC AND SYSTEMIC DATA:
  Age: [___] | Sex: [Male / Female]
  Smoking: [non-smoker / ex-smoker / current: ___ pack-years]
  Diabetes: [no / yes - HbA1c: ___%]
  Systemic conditions: [none / specify]
  Medications: [none / specify]


PERIODONTAL CHARTING SUMMARY:
  Mean PPD: [___] mm | Max PPD: [___] mm
  Sites PPD >=5 mm: [___] | Sites PPD >=6 mm: [___]
  Mean CAL: [___] mm | Max CAL (interdental): [___] mm
  BOP: [___]% | Plaque score: [___]/3 (Silness-Löe index)
  Mobility: [none / Class I: ___ / Class II: ___ / Class III: ___ teeth]
  Furcation: [none / Class I: ___ / Class II: ___ / Class III: ___ teeth]
  Missing teeth: [none / specify]
  Affected teeth: [___] of [___ total remaining] teeth ([___]%)


RADIOGRAPHIC FINDINGS (clinician-derived panoramic summary,
  two independent examiners, ICC reported in manuscript):
  Max bone loss: [___]% of root length
  Bone loss/age ratio: [___]
  Pattern: [horizontal / vertical / combined]
  Vertical defects: [none / present - location: specify]
  Furcation findings: [none / present - location: specify]
  Tooth loss due to periodontitis: [none / ___ teeth]


GRADE MODIFIERS:
  Smoking modifier: [none / <10 cig/day / >=10 cig/day]
  Diabetes modifier: [none / HbA1c <7% / HbA1c >=7%]


COMPLEXITY FACTORS (binary, based on clinician assessment):
  Posterior bite support loss: [yes / no]
  Pathological migration / flaring: [yes / no]
  Masticatory dysfunction: [yes / no]


--- CLASSIFICATION QUESTION ---
Q1. What is the correct periodontal diagnosis per the 2018 AAP/EFP
classification?
    State Stage, Grade, and Extent separately.
    Final decision / Rationale
```

```
--- END OF CASE ---
```

*Note: there is deliberately no "Extent" field in the case form; the model determines Extent itself from the "Affected teeth ... of ... total remaining teeth (___%)" line, applying the 2018 AAP/EFP ≤30%/>30% localized/generalized threshold independently (Section 2.6 of the main manuscript).*

## C. Illustrative Example of Model Output Format

To illustrate that Stage/Grade/Extent extraction from LLM output was a direct, verbatim transcription rather than an interpretive judgement (Section 2.5 of the main manuscript), the box below reproduces one representative, unedited session response in the exact format returned across sessions and models. The example is anonymised (case identifier, model identity, and session number withheld) and reproduced for illustrative purposes only; formatting reflects the model's own raw output.

*"Final decision: Generalized Stage III, Grade C periodontitis. Rationale: Maximum CAL of 7 mm and bone loss at 50% of root length place severity at Stage III (not Stage IV, since no complexity factor — posterior bite support loss, pathological migration, or masticatory dysfunction — is present); the bone-loss/age ratio of 1.09 (≥1.0) indicates Grade C progression, and involvement of 42.86% of teeth (≥30%) defines a generalized extent."*

As shown, each session's response states one unambiguous Stage/Grade/Extent decision in its opening sentence; this stated decision, not the supporting rationale, was transcribed directly into the data-capture workbook.

## D. Full Sample-Size Pilot Rule

This section reproduces, in full, the pre-specified pilot-based rule referenced in Section 2.8 of the main manuscript, which was used alongside the kappa-precision calculation (target κ = 0.40, lower bound 0.20, n ≥ 45; conservative scenario target κ = 0.30, lower bound 0.10, n ≥ 58) to confirm that the minimum planned sample of n = 60 was appropriate before full-scale case accrual began.

A pilot batch of 10 cases (the first 10 consecutively accrued cases, cases 1–10, which were retained in the main analysis; an internal pilot) was classified by all three LLMs under the full study protocol, and Fleiss' kappa was computed across the three models' modal Stage decisions for these 10 cases (the coefficient was mislabeled "Fleiss' W" in the originally submitted version of this file). The pre-specified decision rule was: if Fleiss' kappa ≥ 0.10, the minimum planned sample of n = 60 was retained; if it fell between 0.08 and 0.10, the minimum target was increased to n = 80; and if it was below 0.08 (indicating higher-than-expected between-model variability, requiring greater precision), the minimum target was increased to n = 100. This rule was designed solely as a safeguard that could increase the minimum sample; a low threshold was chosen so that only marked between-model disagreement would trigger enlargement, and meeting the threshold was not taken as evidence of adequate agreement. The observed pilot value was Fleiss' κ = 1.00 (all three models gave the same modal Stage in 10 of 10 cases; 7 Stage III, 3 Stage IV), so the minimum of n = 60 was retained. Case accrual then continued beyond this minimum, consecutively as eligible records became available at the two centres, to a locked total of n = 105, which exceeded the largest minimum (n = 100) that the rule could have imposed under any pilot outcome.

## E. Specification and Code of the Exploratory Bayesian Mixed-Effects Model (added in revision)

This section documents the exploratory Bayesian mixed-effects logistic regression reported in Sections 2.9 and 3.9 of the main manuscript. Outcome: session-level Stage concordance (1 = the session's Stage decision matched the adjudicated reference; 0 = otherwise) for all 945 sessions (105 cases × 3 models × 3 sessions). Fixed effects: model (ChatGPT [reference], Claude, Gemini), reference Stage (Stage III [reference], Stage IV), and center (Center B/Burdur [reference], Center A/Batman). Random effect: intercept per patient (105 levels), accounting for the clustering of nine sessions within each case.

Priors (weakly informative, logit scale): intercept ~ Normal(0, 2.5); each fixed-effect coefficient ~ Normal(0, 2.5); patient random-intercept standard deviation ~ Half-Normal(2.5); random intercepts ~ Normal(0, SD).

Estimation: No-U-Turn Sampler (NUTS) via PyMC 5.28 through the Bambi 0.17 interface; 4 chains; 2000 warm-up (tuning) and 2000 sampling iterations per chain (8000 posterior draws); target acceptance 0.95; random seed 2026. Diagnostics: potential scale reduction factor (R-hat), bulk and tail effective sample size (ESS), and number of divergent transitions, computed with ArviZ. In the reported fits, all R-hat values were 1.00, bulk ESS exceeded 2500 for every parameter, and no divergent transitions occurred.

Reported quantities: posterior median odds ratios (exp of the coefficient posterior) with 95% equal-tailed credible intervals. Three additional specifications were fitted to examine the collinearity of Stage and center: (i) the full model without the Stage term; (ii) the full model without the center term; (iii) the full model with a Stage × center interaction. Results are given in Section 3.9 of the main manuscript.

Python code (analysis performed on the locked session-level dataset; column names: conc, model, stage, center, case):

```
import pandas as pd, numpy as np, bambi as bmb, arviz as az
d = pd.read_csv("sessions.csv")   # one row per session: conc (0/1), model, stage, center, case
d["model"]  = pd.Categorical(d["model"],  ["ChatGPT", "Claude", "Gemini"])
d["stage"]  = pd.Categorical(d["stage"],  ["Stage III", "Stage IV"])
d["center"] = pd.Categorical(d["center"], ["burdur", "batman"])
d["case"]   = d["case"].astype(str)
priors = {"Intercept": bmb.Prior("Normal", mu=0, sigma=2.5),
          "common":    bmb.Prior("Normal", mu=0, sigma=2.5),
          "1|case":    bmb.Prior("Normal", mu=0, sigma=bmb.Prior("HalfNormal", sigma=2.5))}
def fit(formula):
    m = bmb.Model(formula, d, family="bernoulli", priors=priors)
    idata = m.fit(draws=2000, tune=2000, chains=4, target_accept=0.95, random_seed=2026)
    requested = ["model", "stage", "center", "stage:center", "1|case_sigma"]
    available = [v for v in requested if v in idata.posterior]
    print(az.summary(idata, var_names=available, hdi_prob=0.95))
    print("divergences:", int(idata.sample_stats.diverging.sum()))
    for v in ["model", "stage", "center", "stage:center"]:
        if v in idata.posterior:
            for lvl in idata.posterior[v + "_dim"].values:
                orr = np.exp(idata.posterior[v].sel({v + "_dim": lvl}).values.flatten())
                print(v, lvl, "OR = %.2f  95%% CrI [%.2f, %.2f]" % (np.median(orr), *np.percentile(orr, [2.5, 
97.5])))
    return idata
fit("conc ~ model + stage + center + (1|case)")   # full model (Section 3.9)
fit("conc ~ model + center + (1|case)")           # without Stage
fit("conc ~ model + stage + (1|case)")            # without center
fit("conc ~ model + stage * center + (1|case)")   # Stage x center interaction
```
