# Supplementary material for: Staging and Grading Advanced Periodontitis with Large Language Models from Standardized Case Summaries: A Multicenter Concordance and Reproducibility Study
Source: J Clin Med. 2026 Sep 30;15(19):7585. doi: 10.3390/jcm15197585 (PMC13665847; doi:10.3390/jcm15197585)
Supplement: Supplementary file 1 [file jcm-15-07585-s001.zip › File_S5_TRIPODLLM_Checklist_R1.pdf]

SUPPLEMENTARY FILE S5 — TRIPOD-LLM Checklist

Manuscript: Staging and Grading Advanced Periodontitis with Large Language Models from Standardized Case Summaries: A Multicenter Concordance and Reproducibility Study

Authors: Mehmet Kızıltoprak, Mustafa Karaca, Mustafa Özay Uslu

This checklist follows the TRIPOD-LLM reporting guideline for studies using large language models (Gallifant J, Afshar M, Ameen S, et al. The TRIPOD-LLM reporting guideline for studies using large language models. Nat Med. 2025;31(1):60–69. doi:10.1038/s41591-024-03425-5), used here to report the LLM-specific aspects of this study that are not fully captured by STROBE (Supplementary File 1), GRRAS (Supplementary File 2), or STARD-AI (Supplementary File 3).

Applicability filtering: The full TRIPOD-LLM checklist comprises 19 main items / 50 subitems, of which a subset applies to any given study depending on research design and LLM task (the guideline's own modular framework). This study's research design is "LLM evaluation" (E) — assessing existing, off-the-shelf models without development, fine-tuning, or clinical-workflow deployment — and its LLM task is "Classification" (C). Items tagged only for de novo development (M/D), healthcare-deployment (H), or other LLM tasks (QA, IR, DG, SS, MT) are marked Not Applicable (N/A) below and are not required for this study; this filtering follows the checklist's own published modular structure (see Table 1 and Fig. 1 therein).

Status key: Reported = fully addressed in the current manuscript. Partially reported = addressed but could be more explicit. GAP = not currently addressed; author input or a manuscript addition is needed. N/A = not applicable to this study's design/task per the checklist's own modular filtering.

Section	Item	Description	Status	Reported in manuscript / action needed
Title	1	Identify the study as developing, fine-tuning and/or evaluating the performance of an LLM, specifying the task, the target population and the outcome to be predicted.	Reported	*Title page*
Abstract	2	See TRIPOD-LLM for abstracts (Table 3).	Reported	*Abstract*
Introduction — Background	3a	Explain the healthcare context/use case and rationale for developing or evaluating the LLM, including references to existing approaches and models.	Reported	*Introduction*
	3b	Describe the target population and the intended use of the LLM in the context of the care pathway, including intended users in current gold-standard practices.	Reported	*Discussion, Section 4.1 (Limitations) (explicit sentence: clinician-supervised decision support, periodontology-trained user prepares the case summary and reviews LLM output against the complete clinical record; not autonomous/unsupervised use, not for non-specialist personnel without review)*
Introduction — Objectives	4	Specify the study objectives, including whether the study describes LLM development, tuning or validation.	Reported	*Introduction, final paragraph*
Methods — Data	5a	Describe the sources of data for the evaluation dataset and the rationale for using it.	Reported	*Section 2.2*
	5b	Describe the relevant data points and provide a quantitative/qualitative description of the dataset.	Reported	*Section 2.2; Table 1*
	5c	State the date of the oldest and newest item of text used in the evaluation dataset.	Reported	*Section 2.2 (patient records collected/case summaries prepared 8–18 July 2026, following 7 July 2026 ethics approval, preceding the 18–24 July 2026 LLM testing window)*
	5d	Describe data preprocessing and quality checking, including consistency across sources/institutions.	Reported	*Sections 2.2, 2.4, 2.6*
	5e	Describe how missing/imbalanced data were handled and reasons for omitting data.	Reported	*Section 2.2 (exclusions), 2.7 (unclassifiable outputs)*
Methods — Analytical methods	6a	Report the LLM name, version and last date of training.	Reported	Table 2 (name/displayed label/platform/testing window reported; Section 2.4 notes that the displayed label does not identify a verifiable backend

Section	Item	Description	Status	Reported in manuscript / action needed
				version); model training-cutoff dates are not publicly disclosed by the vendors for these versions and could not be independently verified by the authors — stated explicitly as a reporting constraint
	6b	LLM development process (architecture, fine-tuning, alignment). [Design: de novo development/methods only]	N/A	*Not applicable — off-the-shelf models, no development/fine-tuning performed*
	6c	Details of prompt engineering/output generation and inference settings (seed, temperature, max tokens).	Reported	*Section 2.4; Supplementary File 4 (verbatim prompt). Temperature, seed, and other inference settings were not accessible via the consumer web interfaces used (chatgpt.com, claude.ai, gemini.com) and therefore could not be logged; this is stated explicitly as a methodological constraint of testing via consumer deployment rather than API access*
	6d	Specify the initial and postprocessed output of the LLM (e.g., probabilities, classification, unstructured text).	Reported	Sections 2.5 and 2.7 (hard classification; no output probabilities available via consumer interfaces; vote pattern across sessions used as a stability indicator)
	6e	Details/rationale for classification and how thresholds were identified.	Reported	*Section 2.6 (30% Extent threshold); Supplementary File 4*
Methods — LLM output	7b	Report outcome metrics' relevance to the downstream task and correlation to human evaluation, where applicable.	Reported	*Sections 2.3, 2.9*
	7c	Clearly define the outcome, how LLM predictions were calculated, and date of inference for closed-source LLMs.	Reported	*Sections 2.4, 2.7; Table 2*
	7d	If outcome assessment required subjective interpretation, describe assessor qualifications/instructions/agreement.	Reported	Section 2.5 (extraction was verbatim, not interpretive); Section 2.3 (expert qualifications)
	7e	Specify how performance was compared to other LLMs, humans and benchmarks.	Reported	*Sections 3.4–3.6; Discussion vs Round 1 human benchmark*
Methods — Annotation	8a	If annotation was done, report how text was labeled, with guidelines/examples.	Reported	*Section 2.3 (reference-standard classification protocol)*
	8b	Report number of annotators/experts, proportion double-annotated, and inter-annotator agreement.	Reported	*Section 2.3; Section 3.2 (Round 1 kappa)*
	8c	Background/experience of annotators.	Reported	*Section 2.3 (Experts A/B: both board-certified periodontologists, with 7 and 8 years of post-certification clinical experience, respectively; Expert C: third, independent periodontologist with 12 years of post-doctoral experience, who adjudicated the 7 discordant cases not resolved by structured consensus)*
Methods — Prompting	9a	Details on prompt design, curation and selection process.	Reported	*Section 2.4; Supplementary File 4. The system instruction and case-form template were derived directly from the 2018 AAP/EFP criteria (Tonetti MS, Greenwell H, Kornman KS. Staging and grading of periodontitis: Framework and proposal of a new classification and case definition. J Clin Periodontol. 2018;45(Suppl 20):S149–S161), without pilot-testing or iterative refinement on trial cases*

Section	Item	Description	Status	Reported in manuscript / action needed
	9b	What data were used to develop the prompts.	Reported	*Section 2.4. No separate prompt-development dataset was used; the prompt was developed directly from the published 2018 AAP/EFP classification criteria, not from piloted patient cases*
Methods — Compute	12	Report compute or proxies thereof (time, machines, cost, inference time). [Design: E applicable]	Reported	*Not reported: compute metrics (inference time, cost, FLOPs) are not exposed by the consumer web interfaces used and could not be logged; stated explicitly as a constraint of consumer-deployment testing rather than an omission*
Methods — Ethical approval	13	Name the ethics committee that approved the study and describe consent or waiver.	Reported	*Section 2.1; Declarations — Ethics approval / Consent to participate*
Methods — Open science	14a	Source of funding and role of funders.	Reported	*Declarations — Funding*
	14b	Conflicts of interest and financial disclosures for all authors.	Reported	*Declarations — Competing interests*
	14e	Availability of the study data.	Reported	*Declarations — Availability of data and materials*
	14f	Availability of code to reproduce study results.	Reported	*Declarations — Code availability (analysis code/scripts available from the corresponding author upon reasonable request, on the same basis as the case-level dataset)*
Results — Participants	16a	Flow of participants through the study.	Reported	*Fig. 1; Section 3.1*
	16b	Characteristics overall and for each data source/setting.	Reported	*Table 1; Section 3.1*
	16c	Comparison of clinical variables between development/evaluation splits.	N/A	*Not applicable — no development/training split; pure evaluation design*
	16d	Number of participants and outcome events in each analysis.	Reported	*Section 3.1; Tables 2–5*
Results — Performance	17	Report LLM performance per prespecified metrics and/or human evaluation.	Reported	*Sections 3.4–3.9; Tables 2–5*
Results — LLM updating	18	If applicable, report results from any LLM updating.	Reported	*Section 2.4* (explicitly: no change in the displayed model label during the study period; backend model identity could not be verified, Section 4.1)
Discussion — Interpretation	19a	Overall interpretation of results, including fairness considerations.	Reported	*Discussion; residual centre effect explicitly reframed as an equity/fairness consideration for uneven performance across clinical settings (Discussion)*
Discussion — Limitations	19b	Discuss limitations and their effects on bias, statistical uncertainty and generalizability.	Reported	*Section 4.1*
Discussion — Usability of LLM in context	19c	Known challenges in using data for this task/domain (representation, missingness, harmonization, bias).	Reported	*Section 4.1 (spectrum bias; single-country, two-centre representativeness limitation added)*
	19d	Define intended use for the implementation evaluated, including input, end-user and level of autonomy/human oversight.	Reported	*Discussion, Section 4.1 (Limitations) (explicit sentence: clinician-supervised decision support, periodontology-trained user, not autonomous diagnosis)*
	19e	How poor-quality/unavailable input data should be assessed/handled in implementation.	Reported	*Section 4.1 (stated: all cases had complete data meeting eligibility criteria; behaviour with incomplete/missing input not tested*

Section	Item	Description	Status	Reported in manuscript / action needed
				and remains unknown)
	19f	Whether users must interact with input/output handling and what expertise is required.	Reported	*Discussion, Section 4.1 (Limitations) (explicit sentence: periodontology-trained user required; not intended for non-specialist personnel without review)*
	19g	Next steps for future research regarding applicability/generalizability.	Reported	*Discussion (future research directions paragraph); Section 4.1*

Page numbers refer to the main manuscript (Word/PDF pagination) and may shift with journal typesetting; section numbers are stable and should be used as the primary cross-reference. TRIPOD-LLM is a living guideline; the current version should be confirmed at https://tripod-llm.vercel.app/ before final submission, as items may be updated after this checklist was completed.
